# Histopathologic Findings in Sleeve Gastrectomy Specimens: Is Routine Pathological Examination Always Necessary?

**DOI:** 10.30699/ijp.2025.2032599.3316

**Published:** 2025-11-12

**Authors:** Safa Nourani, Elham Mirzaian, Mahdis Khazaeli Najafabadi, Seyed Mohammad Tavangar, Salma Sefidbakht, Abdolreza Pazouki

**Affiliations:** 1 Department of Pathology, Dr Shariati Hospital, School of Medicine, Tehran University of Medical Sciences, Tehran, Iran; 2 Laparoscopic Surgery Department of General Surgery, School of Medicine Minimally Invasive Surgery Research Center, Iran University of Medical Sciences, Tehran, Iran

**Keywords:** Sleeve gastrectomy, Histopathological findings, clinical characteristics

## Abstract

**Background & Objective:**

There are limited findings regarding histopathological changes in sleeve gastrectomy samples and their relationship with preoperative clinical and histopathological characteristics. The present study aims to assess histopathological findings in sleeve gastrectomy samples and identify the main determinants of these changes.

**Methods:**

This cross-sectional study retrospectively reviewed demographic, preoperative clinical, histological, and endoscopic findings of 258 patients who underwent laparoscopic sleeve gastrectomy surgery. Postoperative pathological findings were also evaluated.

**Results:**

Microscopic examination revealed pathological findings in 212 samples (82.2%). The most common histopathological finding reported in patients was chronic gastritis, present in approximately 67.1% of cases, followed by active gastritis in 13.6%. Additionally, 19.0% of patients tested positive for helicobacter pylori infection. A significant association was found between the history of hyperlipidemia and helicobacter pylori positivity (p = 0.039). Before surgery, 80 patients (41.7%) had normal endoscopic results, while at least one significant abnormal finding was observed in 58.3% of cases. However, there was no significant relationship between preoperative endoscopic findings and histopathological changes after surgery in almost all examined patients.

**Conclusion:**

Histopathological examination of sleeve gastrectomy samples reveals a high prevalence of abnormal findings, including active gastritis, Helicobacter pylori infection, intestinal metaplasia, and dysplasia requiring therapeutic management. However, tracking these changes in biopsy samples obtained from endoscopy before surgery may not be sufficient to predict the histopathological findings after sleeve gastrectomy.

## Introduction

Obesity is a rapidly growing, life-threatening global epidemic that poses significant public health challenges (1). In the Middle East, the prevalence of overweight and obesity is particularly high, affecting approximately 74% to 86% of women and 69% to 77% of men (2). Morbid obesity significantly increases the risk of numerous comorbidities, including type 2 diabetes mellitus, hypertension, stroke, cardiovascular disease, dyslipidemia, osteoarthritis, asthma, and various malignancies (3).

Achieving sustained weight loss through lifestyle modification and pharmacologic therapy remains difficult, especially in individuals with severe obesity and obesity-related comorbid conditions. As a result, bariatric surgery is now considered the most effective and durable treatment for morbid obesity (4). Since the mid-20th century, various surgical techniques have been introduced, with laparoscopic Roux-en-Y gastric bypass and laparoscopic sleeve gastrectomy emerging as the two most commonly performed procedures (5,6).

Sleeve gastrectomy involves the resection of a substantial portion of the stomach, particularly the gastric fundus and corpus along the greater curvature, resulting in the removal of approximately 60% of the stomach. This creates a tubular gastric remnant with a volume of 60 to 130 mL, effectively restricting caloric intake (7). The procedure offers several advantages, including significant weight loss, a relatively short recovery period, favorable safety profile, and low mortality rate (8).

Unlike other bariatric procedures, sleeve gastrectomy entails actual gastric resection, raising the question of whether routine histopathological evaluation of resected specimens is necessary. Currently, there are no universally accepted guidelines regarding the pathological assessment of these specimens. Nevertheless, many medical centers routinely submit sleeve gastrectomy specimens for comprehensive histopathological examination. This practice offers a unique opportunity to identify underlying gastric pathology in morbidly obese patients.

However, due to variations in diagnostic criteria, institutional protocols, and population demographics, the existing literature on histopathological findings in sleeve gastrectomy specimens is heterogeneous and sometimes contradictory. While some studies have reported a relatively high incidence of unexpected histopathological findings requiring further clinical follow-up (11–13), others have suggested that the majority of these specimens are unremarkable and that routine histopathological analysis may be unnecessary (14).

Given the limited number of studies conducted in our country and the ongoing debate regarding the value of routine specimen analysis, this study was designed to investigate the histopathological findings in sleeve gastrectomy specimens from our center.

## Materials and Methods

### Patient Selection and Study Design

This retrospective cross-sectional study evaluated 258 laparoscopic sleeve gastrectomy specimens collected between 2019 and 2021 from patients who underwent surgery at Shariati and Atieh hospitals in Tehran, Iran. Patient records were retrieved from the hospitals’ electronic databases, and demographic and clinical information—including age, sex, body mass index (BMI), and relevant medical history (eg, diabetes mellitus, hypertension, hyperlipidemia, fatty liver disease, and hypothyroidism)—was recorded. Additionally, preoperative endoscopy findings and operative reports were reviewed.

Histopathological assessment was performed by examining slides stained with hematoxylin and eosin (H&E) and Giemsa, along with corresponding paraffin-embedded tissue blocks. All evaluations were conducted by a pathologist blinded to the patients’ clinical and demographic data. The following abnormal histological features were recorded: chronic gastritis (active or inactive), intestinal metaplasia, dysplasia, polyps, atrophy, reactive changes, malignancy, and *Helicobacter pylori* infection. Macroscopic findings from gross examination were also obtained from pathology reports.

### Sample Size Calculation

The entire population of sleeve gastrectomy specimens from the selected hospitals between 2019 and 2021 was included. The required sample size was calculated based on data from Komaei et al (4), who reported a 63.5% prevalence of chronic gastritis, the most frequent abnormal histopathological finding. Using Cochran’s formula for sample size estimation and this prevalence rate, a minimum of 220 cases was determined to be necessary. Ultimately, 258 specimens were analyzed.

### Statistical Analysis

Statistical analysis was performed using SPSS software (version 23.0; IBM Corp). Data were analyzed descriptively and analytically. Quantitative variables were expressed as mean ± standard deviation (SD), and qualitative variables were reported as frequencies and percentages.

The Kolmogorov–Smirnov test was used to assess the normality of numerical data distributions. For inferential analysis, the Mann–Whitney U test was applied to compare continuous variables that did not follow a normal distribution (such as age and BMI). Categorical variables were compared using the chi-square test. Statistical associations and relationships were described using appropriate terminology, and a *P* value of less than 0.05 was considered statistically significant.

## Results

A total of 258 sleeve gastrectomy specimens were evaluated histopathologically. The mean age of patients was 37.17 ± 11.00 years (range: 12–74 years), and 72.5% were female. Regarding anthropometric data, the mean weight was 119.05 ± 20.06 kg, the mean height was 165.86 ± 9.04 cm, and the mean body mass index (BMI) was 43.11 ± 5.91 kg/m². Among the patients, 19.0% had a BMI between 35 and 40 kg/m², and 79.5% had a BMI greater than 40 kg/m².

The most common comorbid condition was fatty liver disease, observed in 27.5% of patients, followed by hypertension (19.0%) and hypothyroidism (18.2%).

No abnormal findings were reported in macroscopic examinations. However, microscopic histopathological abnormalities were identified in 212 specimens (82.2%), while 46 specimens (17.8%) showed no significant pathological changes. The frequency of each histopathological finding is summarized in Table 1. The most common finding was chronic gastritis (67.1%), followed by active gastritis (13.6%). *Helicobacter pylori* infection was detected in 49 specimens (19.0%).

A significant association was found between a history of hyperlipidemia and *H. pylori* positivity (*P* = 0.039). However, no significant relationship was observed between patients’ age or BMI and the presence of any specific histopathological findings.

Preoperative endoscopy results were available for 192 patients (74.4%). Of these, 80 patients (41.7%) had normal findings, while 112 patients (58.3%) showed at least one significant abnormality. The most common endoscopic abnormalities were hiatal hernia (28.6%), esophagitis (22.9%), erosive gastritis (19.8%), and gastric ulcer (5.2%). Preoperative *H. pylori* positivity was noted in 57.3% of patients.

Preoperative histological evaluations were performed in 114 patients (44.2%). Among these, 6.1% had normal histology, while 53.5% had chronic gastritis, 29.8% had active gastritis, 0.9% had reactive gastropathy, and 9.6% had intestinal metaplasia.

Analysis of the relationship between preoperative endoscopic findings and postoperative histology revealed that among patients with normal endoscopy, 73.8% had chronic gastritis, 10.0% had active gastritis, and 16.3% were positive for *H. pylori*. No significant differences in postoperative histological findings were observed between patients with normal versus abnormal preoperative endoscopic findings (Table 2).

Among those with normal preoperative histology, 6.1% developed postoperative chronic gastritis. Notably, all patients with preoperative intestinal metaplasia also demonstrated postoperative intestinal metaplasia. This association was statistically significant (*P* = 0.001), suggesting persistence of the condition (Table 3). However, no significant differences were observed in postoperative histology between patients with and without preoperative *H. pylori* infection.

**Table 1:** The frequency of histopathological findings in the examined samples

Histopathological findings	Frequency (%)
Chronic gastritis	173 (67.1)
Active gastritis	35 (13.6)
Congestion	10 (3.9)
Intestinal metaplasia	6 (2.3)
Dysplasia	1 (0.4)
Reactive gastropathy	1 (0.4)
Heterotopy	1 (0.4)
Erosion	1 (0.4)
Reactive atypia	1 (0.4)

**Table 2:** The association between preoperative endoscopic findings and postoperative histological results

Preoperative Endoscopic findings	postoperative histological results			
	Chronic gastritis	Active gastritis	Intestinal metaplasia	Helicobacter pylori
Normal	59 (73.8)	8 (10.0)	0 (0.0)	13 (16.3)
Abnormal	69 (61.7)	21 (18.8)	4 (3.6)	25 (22.3)
P value	0.078	0.095	0.088	0.298

**Table 3:** The association between preoperative histologic findings and postoperative histological results

preoperative histologic findings	postoperative histological results		
	Chronic gastritis	Active gastritis	Intestinal metaplasia
Normal	5 (6.1)	0 (0.0)	0 (0.0)
Chronic gastritis	46 (56.1)	6 (37.5)	0 (0.0)
Active gastritis	23 (28.0)	7 (43.8)	0 (0.0)
Intestinal metaplasia	8 (9.8)	3 (18.8)	2 (100)
P value	0.518	0.287	0.001

## Discussion

In recent years, it has been proposed that the histopathological evaluation of sleeve gastrectomy specimens should be performed selectively, based on suspicious lesions identified during preoperative endoscopy or intraoperative findings, to ensure cost-effectiveness. This study aimed to assess the histopathological changes in sleeve gastrectomy specimens and explore their associations with demographic characteristics and underlying comorbidities in 258 patients undergoing bariatric surgery.

Our results revealed that abnormal histopathological findings were present in a significant proportion of cases (82.2%). The most frequent pathology was chronic gastritis, observed in 67.1% of specimens, followed by active gastritis in 13.6%, indicating that a total of 80.7% of patients had some form of gastritis. These findings are consistent with multiple studies, although the reported prevalence of chronic gastritis varies widely. For instance, Komaei et al. in Italy reported chronic gastritis in 63.7% of specimens (4), while Adali et al. in Turkey noted chronic gastritis in 100% of cases (inactive in 73%, active in 27%) (15). Other studies from Turkey and Jordan have reported prevalence rates of 73.9% and 93.3%, respectively (16,17), while an investigation from Saudi Arabia noted a rate of 83.4%, with 22.3% showing concurrent *Helicobacter pylori* (H. pylori) infection (18).

In contrast, studies from Qatar, Canada, and the United States have documented lower prevalence rates. For example, Safaan et al. found that 52% of sleeve gastrectomy samples had normal histology, with 33% showing inactive chronic gastritis and 6.8% active gastritis (2). Di Palma et al. in Canada reported 51% normal histology and 25.7% chronic gastritis (5), while Clapp in the U.S. observed abnormal histopathology in 49.7% of samples, with chronic gastritis being the most frequent finding (19). These discrepancies may be attributed to geographic variation in *H. pylori* prevalence, dietary habits, and diagnostic criteria.

The high prevalence of chronic gastritis among morbidly obese patients has led to the hypothesis of a distinct category, obesity-related gastritis, which suggests that obesity itself may contribute to gastritis independent of *H. pylori* infection (20). The precise mechanism, however, remains unclear.

Other histological abnormalities, such as mucosal congestion, heterotopia, and reactive atypia, were infrequent, each occurring in fewer than 5% of cases. Similarly, low frequencies of premalignant lesions, intestinal metaplasia (2.3%) and dysplasia (0.4%), were detected. These results align with previous studies reporting variable rates of intestinal metaplasia and dysplasia: Komaei et al. reported 3.8% and 1.1%, respectively (4), while Safaan et al. found intestinal metaplasia in 1.4% with no dysplasia (2). A Saudi Arabian study reported a 1% prevalence of intestinal metaplasia (18), and Kopach et al. in the U.S. noted 1.5% metaplasia without dysplasia (21).

Chronic gastritis has been implicated in the progression to atrophic gastritis, intestinal metaplasia, and ultimately gastric dysplasia and carcinoma through cumulative genetic and epigenetic changes (22,23). Therefore, the presence of intestinal metaplasia, although low in frequency, remains clinically significant and warrants surveillance.

In our study, *H. pylori* infection was detected in 19% of sleeve gastrectomy specimens. This finding is comparable to that of Clapp et al. (18%) (19) and a study from Saudi Arabia (22.3%) (18). However, higher prevalence rates have been reported in Turkey (27%–41.7%) and Qatar (41%) (2,7,16). These variations likely reflect differences in socioeconomic status, hygiene, diet, and regional public health policies. It is also important to consider that in many centers, *H. pylori* eradication is performed preoperatively, which may affect postoperative detection rates and lead to underestimation.

Indeed, we observed a notable reduction in *H. pylori* prevalence from 57.3% in preoperative endoscopy samples to 19.0% in sleeve gastrectomy specimens, likely due to successful eradication therapy administered before surgery.

Our analysis did not find a statistically significant correlation between histopathological findings and age or BMI. This aligns with the findings of Di Palma et al. (5) and Safaan et al. (2), but contrasts with Adali et al., who found a significant inverse relationship between BMI and intestinal metaplasia prevalence (15). Komaei et al. also found no association with BMI or sex but did report a significant relationship between older age and premalignant lesions (4). In our study, although patients with intestinal metaplasia were, on average, six years older than those without, the difference did not reach statistical significance. A larger sample size may be needed to detect such associations.

We identified a significant relationship between *H. pylori* positivity and hyperlipidemia. Although this finding lacks a clearly defined biological explanation, emerging research supports a possible link. Ansari et al. reported elevated lipid parameters in patients with *H. pylori*-associated gastritis (25). Similarly, Aarabi et al. found increased triglyceride levels and total cholesterol/HDL ratios in *H. pylori*-positive patients (26), and Kim et al. observed higher LDL-C and lower HDL-C in seropositive individuals (27). Haeri et al. and Sun et al. also noted alterations in lipid profiles associated with *H. pylori* infection (28,29), and evidence suggests that eradication may improve lipid ratios (30). These findings warrant further investigation.

We found no statistically significant association between most endoscopic abnormalities and histopathological outcomes. However, the presence of intestinal metaplasia before surgery was significantly associated with its postoperative persistence, underscoring its clinical relevance. Overall, our data suggest that endoscopy alone may not reliably predict underlying gastric pathology.

One of the key challenges in interpreting data across studies is the variability in histopathological classification systems and diagnostic thresholds used by pathologists in different regions. This lack of standardization underscores the need for unified pathological criteria and reporting protocols to enhance inter-center comparability and improve patient care.

## Conclusion

Histopathological examination of sleeve gastrectomy specimens revealed a high prevalence of abnormal findings, including lesions requiring treatment or surveillance, such as active gastritis, *Helicobacter pylori* infection, intestinal metaplasia, and dysplasia. Given these findings, routine histopathological evaluation of sleeve gastrectomy specimens appears warranted, regardless of geographic region or preoperative endoscopic results. Notably, preoperative biopsy and endoscopic assessments may fail to detect clinically significant pathology. Therefore, a comprehensive histopathological review of resected gastric tissue can contribute meaningfully to patient management and follow-up strategies.

## Data Availability

All data generated during the study are included in this article. Further enquiries can be directed at the corresponding author.
